# The Use of Ethanol Sclerotherapy To Treat a Large Cervical Lymphocele

**DOI:** 10.7759/cureus.33043

**Published:** 2022-12-28

**Authors:** Timothy B Shaver, Muhammad El Shatanofy, Weston Niermeyer, Arjun Joshi

**Affiliations:** 1 Otolaryngology - Head and Neck Surgery, George Washington University School of Medicine and Health Sciences, Washington, D.C., USA; 2 Division of Otolaryngology - Head and Neck Surgery, George Washington University School of Medicine and Health Sciences, Washington, D.C., USA

**Keywords:** ultrasound, neck mass, sclerotherapy, ethanol, cervical lymphocele

## Abstract

Cervical lymphoceles are atypical lymphatic accumulations that develop within the subcutaneous tissue of the neck. While these accumulations have traditionally been removed via surgical excision, sclerotherapy has recently emerged as a reasonable option to prevent injury to surrounding vascular, neurologic, and pulmonary structures. The purpose of this case report is to describe the efficacy of ethanol sclerotherapy for a cervical lymphocele refractory to surgical embolization. We present the case of a 70-year-old male with a large cervical lymphocele that was initially treated with surgical embolization. The mass rapidly reaccumulated within two weeks and the patient subsequently underwent ethanol sclerotherapy with no evidence of re-accumulation after 18 months. This case highlights the utility of ethanol due to its better side-effect profile, widespread availability, and cheaper cost when compared to better-described agents.

## Introduction

Cervical lymphoceles are lymphatic accumulations composed of dilated cystic spaces separated by a complex network of connective tissue stroma, lymphoid follicles, and infiltrates [1]. Given the lack of discrete margins found in these tumors, surgical resection can often be difficult and increase the risk of injury to surrounding structures such as the great vessels and lungs [2]. Several case reports have described the use of sclerotherapy as an alternative to surgical excision for cervical lymphoceles. Bleomycin, picibanil (OK-432), and talc have all been described as potential sclerosing agents in the head and neck [3,4]. However, research is limited on the use of ethanol as a sclerosing agent for cervical lymphoceles.

The proposed mechanism for ethanol as a sclerosing agent is through dehydration and denaturation of proteins, leading to thrombus formation and ischemia of the blood vessels supplying the lymphocele [5]. Ethanol has emerged as a favorable sclerosing agent option due to its better side-effect profile, widespread availability, and cheaper cost when compared to better-described agents such as OK-432 [5]. The purpose of this case report is to describe the effectiveness, technical considerations, and safety of ethanol sclerotherapy for the treatment of a large cervical lymphocele, for which medical literature is lacking.

## Case presentation

**Figure 1:**
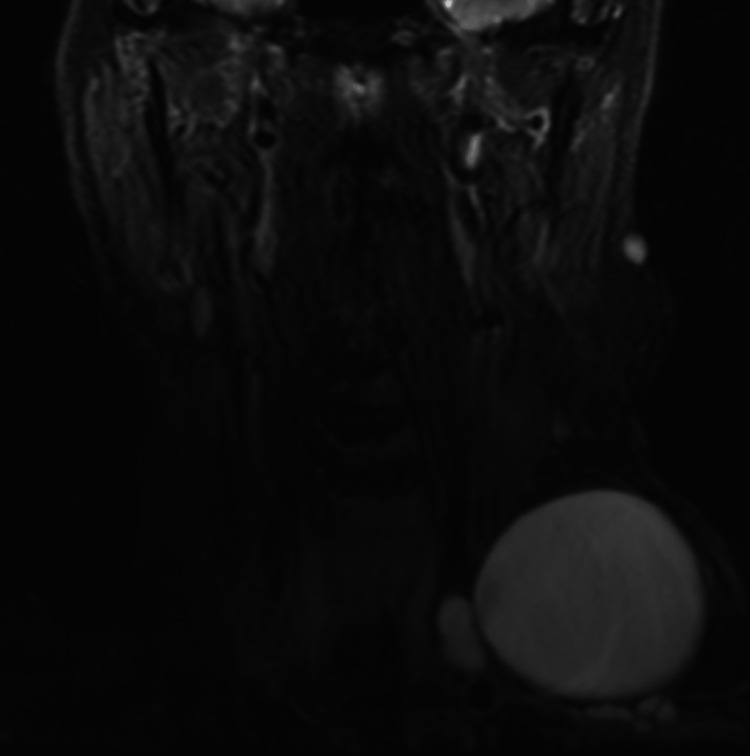
Coronal MRI of head and neck T2 coronal MRI of patient's head and neck area. There is a sizeable left-sided neck mass that is hyperintense on T2 with serpiginous channels located just deep to the dominant mass, suggestive of a cervical lymphocele.

**Figure 2:**
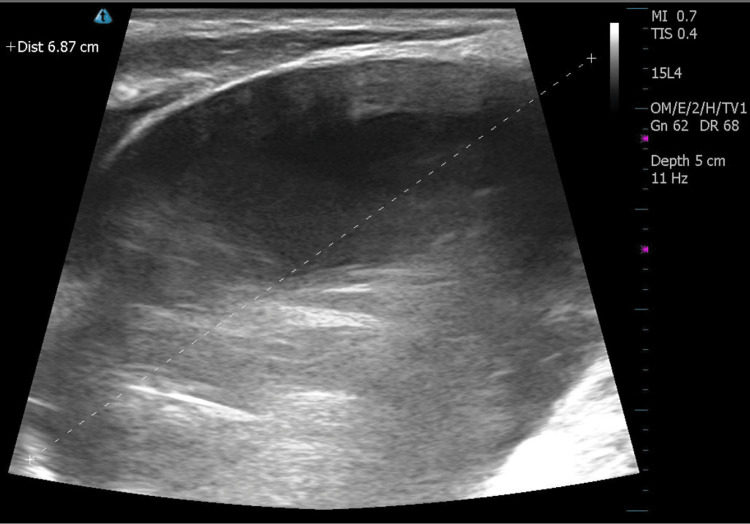
Two-dimensional ultrasound (2DUS) of patient's cervical lymphocele prior to sclerotherapy Two-dimensional ultrasound (2DUS) of the patient’s left neck 16 days following embolization of cervical lymphocele. Transverse orientation shows a large, hypoechoic and fluid-filled mass measuring 6.87 cm.

**Figure 3:**
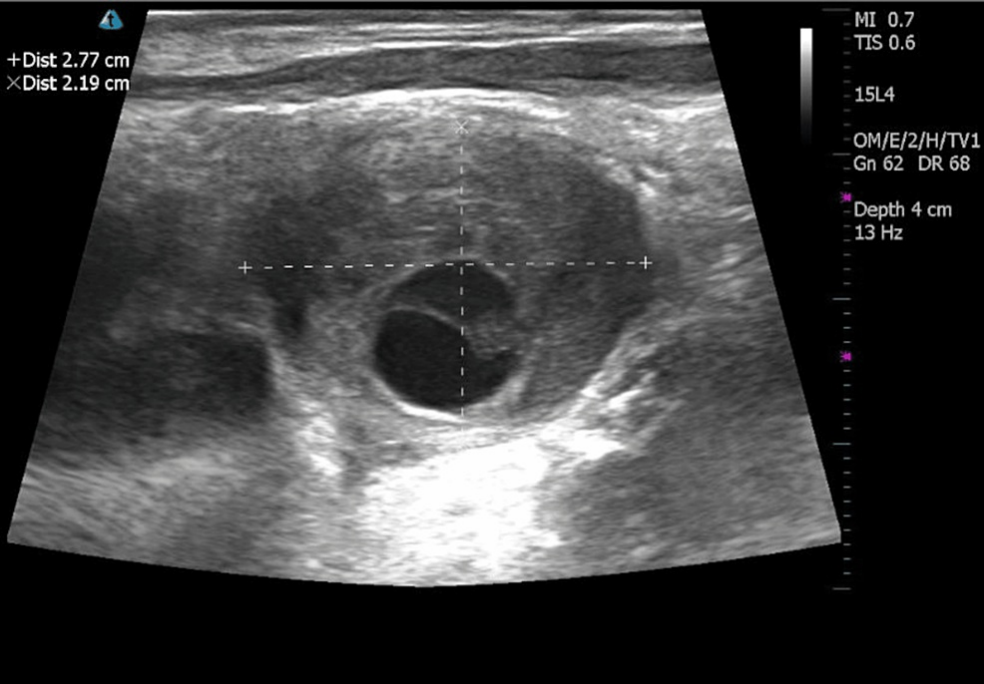
Two-dimensional ultrasound (2DUS) 13 months after sclerotherapy Two-dimensional ultrasound (2DUS) of patient’s left neck at his most recent follow-up visit, 13 months following sclerotherapy. The ultrasound image shows a significantly reduced left cervical lymphocele compared to pre-sclerotherapy measurements (Figure 2). There is a central cystic component with surrounding sclerosis secondary to ethanol therapy. This measures 2.77cm transversely (horizontal line) and 2.19 cm anteroposteriorly (vertical line).

At the initial presentation to an otolaryngology clinic, the patient was an early 70-year-old male with a three-year history of a stable left-sided neck mass. In-office ultrasound was performed at his initial presentation which demonstrated a 6 x 3.5 cm cystic mass in the left neck, thought to represent a lymphangioma or cystic schwannoma at that time. An MRI of the patient’s face, neck, and orbit (F/N/O) was ordered and completed one week later, demonstrating a 6.5 cm cystic mass that projected along the anterior aspect of the left neck and revealed serpiginous channels extending posteriorly into the mediastinum, suggestive of a lymphocele (Figure 1). The patient was referred to an interventional radiologist for embolization which occurred four months after his initial presentation. This included embolizing the entire visualized upper thoracic duct with onyx and interlock coils. Within two weeks of the embolization, the patient noted reaccumulation of the mass. He was seen back in the senior author's practice and subsequent ultrasound identified re-accumulation of his cervical lymphocele, measuring approximately 6.87 cm (Figure 2). Needle aspiration with subsequent ethanol sclerotherapy was recommended. One week later, the patient underwent in-office sclerotherapy where 110 cc of serosanguineous aspirate was withdrawn and 3 cc of 97% ethanol were instilled into the lesion. Two weeks following the first sclerotherapy, the patient returned and a second sclerotherapy treatment was performed in which 90 cc of serosanguineous aspirate was withdrawn and 10 cc of 97% ethanol solution was instilled. Three months later, the patient presented for reassessment and an in-office ultrasound showed a significant reduction in size at 4.8 x 3 x 4.3 cm. On his most recent presentation, 18 months following his initial presentation and 13 months following sclerotherapy, the patient reported a significant reduction in the visual size of the mass, with measurements on in-office ultrasound showing a 2.8 x 2.2 x 2.9 cm mass (Figure 3).

## Discussion

Sclerotherapy is a less invasive alternative to surgical excision for the treatment of cervical lymphoceles and other benign neck masses. While most studies have explored the use of agents such as bleomycin and OK-432, research is limited on the use of ethanol, a widely available and relatively cheaper agent, for the treatment of cervical lymphoceles. In this case review, we described the use of ethanol for the treatment of a large cervical lymphocele, refractory to embolization performed by interventional radiology. The patient received two injections of 97% ethanol with a gradual, yet significant, reduction in the size of his cervical mass over the course of 18 months.

In a retrospective review of 32 patients who underwent percutaneous ethanol sclerotherapy for postoperative lymphoceles, ethanol sclerotherapy permitted the resolution of lymphoceles in 94% of patients [7]. No patients in this study experienced major complications. Fewer than 10% of patients experienced minor complications such as catheter-related infections or catheter dislodgement that required repeated drainage procedures. As with most studies investigating the success, recurrence, and complication rates of patients treated with ethanol sclerotherapy, this study was limited by its small sample size of only 32 patients. Additionally, this study investigated lymphoceles at various anatomical sites, none of which included the cervical region. This presents the question of whether sclerotherapy for cervical lymphoceles can offer similar success rates.

In the case presented in this study, ethanol sclerotherapy resulted in a significant reduction in the size of a left inferior neck lymphocele after only two sclerotherapy sessions. The patient denied any complications immediately post-injection or in the months to follow. The patient experienced over an 80% reduction in the size of the mass after an initial attempt at embolization had resulted in recurrence. The patient was followed for over 18 months, comparable to the duration of monitoring described by prior studies investigating the use of ethanol sclerotherapy [6,7].

There are multiple advantages to using ethanol as an agent for sclerotherapy. First, ethanol is widely available and cost effective, which can facilitate the use of ethanol ablation in the outpatient setting. Additionally, ethanol may be more effective than better-studied agents such as OK-432 for the ablation of other head and neck masses such as branchial cleft cysts [8]. In a retrospective chart review of 22 patients who had undergone ethanol ablation for a second branchial cleft cyst, therapeutic success was achieved in all patients [8]. In this study, the injected volume was 50-80% of the volume of fluid aspirated. In the case presented in this study, only 3-11% of the volume aspirated from the cervical lymphocele was replaced. Future work should explore the optimal volume of aspirated material that should be replaced by ethanol sclerotherapy.

Although the patient experienced a significant reduction in the size of his cervical lymphocele after only two treatments of ethanol sclerotherapy, we are unable to generalize our results due to this being only one case. Additionally, ethanol sclerotherapy was performed after the initial treatment with embolization had failed. It is possible that the prior attempt at embolization may have facilitated subsequent attempts at ethanol ablation. Additionally, the amount of ethanol that was injected was based on the senior author's experience with sclerotherapy for other head and neck masses. Although the patient has significantly benefited from these procedures, future work should explore the volume of ethanol required to achieve optimal resolution of lymphoceles as well as the ideal number and duration between treatments without causing toxicity. Little work has been done to evaluate the threshold of ethanol toxicity in sclerotherapy. However, one study identified a dosage greater than 0.24 mL/kg to be predictive of systemic toxic effects [9]. Further studies, such as large, randomized control trials, should also compare the success, recurrence, and complication rates of patients treated with various sclerotherapy agents.

## Conclusions

Ethanol is a widely available and effective sclerosing agent in the management of lymphoceles. While its utility has previously been described in the management of lymphoceles throughout the body, its use for cervical lymphoceles remains unclear in the existing literature. The purpose of this case presentation is to demonstrate the success of ethanol sclerotherapy in the management of a cervical lymphocele refractory to surgical embolization. We also highlight the safety of using ethanol in scenarios where surgical excision may not be feasible, such as when the lymphoceles encroach on important vascular, neurologic, or pulmonary structures. Future randomized control trials are required to directly compare the success and recurrence rates of surgery, embolization, and sclerotherapy. Additionally, future work is required to elucidate the optimal volume of aspirate and concentrations of ethanol required to treat cervical lymphoceles.

## References

[1] Hamilton BE, Nesbit GM, Gross N, Andersen P, Sauer D, Harnsberger HR (2011). Characteristic imaging findings in lymphoceles of the head and neck. AJR Am J Roentgenol.

[2] Kim JH (2014). Ultrasound-guided sclerotherapy for benign non-thyroid cystic mass in the neck. Ultrasonography.

[3] Hekiert A, Newman J, Sargent R, Weinstein G (2007). Spontaneous cervical lymphocele. Head Neck.

[4] Qureishi A, Silva P, Lamyman A, Cox G (2012). Cervical lymphocoele: a simple solution for a complicated problem. Ann R Coll Surg Engl.

[5] Roh JL, Park CI (2008). OK-432 sclerotherapy of cervical chylous lymphocele after neck dissection. Laryngoscope.

[6] Akhan O, Karcaaltincaba M, Ozmen MN, Akinci D, Karcaaltincaba D, Ayhan A (2007). Percutaneous transcatheter ethanol sclerotherapy and catheter drainage of postoperative pelvic lymphoceles. Cardiovasc Intervent Radiol.

[7] Zuckerman DA, Yeager TD (1997). Percutaneous ethanol sclerotherapy of postoperative lymphoceles. AJR Am J Roentgenol.

[8] Ha EJ, Baek SM, Baek JH, Shin SY, Han M, Kim CH (2017). Efficacy and safety of ethanol ablation for branchial cleft cysts. AJNR Am J Neuroradiol.

[9] Bisdorff A, Mazighi M, Saint-Maurice JP, Chapot R, Lukaszewicz AC, Houdart E (2011). Ethanol threshold doses for systemic complications during sclerotherapy of superficial venous malformations: a retrospective study. Neuroradiology.

